# Mid‐term Results of Subtalar Arthroereisis with Talar‐Fit Implant in Pediatric Flexible Flatfoot and Identifying the Effects of Adjunctive Procedures and Risk Factors for Sinus Tarsi Pain

**DOI:** 10.1111/os.12864

**Published:** 2020-12-17

**Authors:** Sen Wang, Li Chen, Jian Yu, Chao Zhang, Jia‐zhang Huang, Xu Wang, Xin Ma

**Affiliations:** ^1^ Department of Orthopaedics, Huashan Hospital Fudan University Shanghai China; ^2^ Department of Orthopaedics Huashan Hospital North, Fudan University Shanghai China

**Keywords:** Flexible flatfoot, Risk factor, Subtalar arthroereisis, Surgery

## Abstract

**Objectives:**

To (i) report the mid‐term outcomes of subtalar arthroereisis using Talar‐Fit implant for the treatment of flexible flatfoot patients; (ii) compare clinical and radiographic outcomes between arthroereisis with and without adjunctive operative procedures to investigate the effects of adjuncts on the outcomes; and (iii) analyze the risk factors associated with sinus tarsi pain, which is the most common postoperative complication of arthroereisis.

**Methods:**

Thirty‐one flexible flatfoot children and adolescents (46 feet) treated with subtalar arthroereisis using Talar‐Fit implant from June 2014 to May 2019 were retrospectively analyzed. The feet were divided into four treatment groups: (i) arthroereisis alone, (ii) arthroereisis with gastrocnemius recession, (iii) arthroereisis with Kidner procedure, and (iv) arthroereisis with gastrocnemius recession and Kidner procedure. Clinical function was evaluated based on the American Orthopaedic Foot and Ankle Society (AOFAS) ankle and hindfoot score. The following angles were measured for radiographic evaluation: talar‐first metatarsal angle, calcaneal pitch angle, and talar declination angle on the lateral view; and talar‐first metatarsal angle, talocalcaneal angle, and anteroposterior talonavicular coverage angle on the anteroposterior (AP) view. The paired Student's *t*‐test was used to compare the pre‐ and postoperative angular measurements and AOFAS scores. The Wilcoxon rank‐sum test was undertaken to determine the outcome differences among four treatment groups. Multivariate logistic regression analysis was used to analyze risk factors for sinus tarsi pain. *P* value <0.05 is considered statistically significant.

**Results:**

The mean follow‐up of the feet was 32.8 months (range, 10–71 months). The mean AOFAS score significantly improved from 55.5 ± 14.5 preoperatively to 86.3 ± 9.9 (*P* < 0.001). Comparison of radiographic outcomes showed that the lateral talar‐first metatarsal angle decreased by a mean of 19.1° ± 11.9° (*P* < 0.001), the calcaneal pitch angle increased by a mean of 5.4° ± 3.4° (*P* < 0.001), the talar declination angle decreased by a mean of 14.8° ± 9.9° (*P* < 0.001), the AP talar‐first metatarsal angle decreased by a mean of 15.6° ± 10.3° (*P* < 0.001), the AP talocalcaneal angle decreased by a mean of 7.2° ± 8.3° (*P* = 0.001), and the AP talonavicular coverage angle decreased by a mean of 20.4° ± 9.0° (*P* < 0.001). There were no statistically significant differences with regard to AOFAS score and all angle measurements on both the AP and lateral views among the four treatment groups. There was one dislocation case caused by a fall 6 weeks after surgery, which was treated nonoperatively. The incidence of sinus tarsi pain was 13% and logistic regression analysis indicated that patients with a longer distance from the tail end of the implant to the lateral calcaneal wall had 38.8% greater odds of developing sinus tarsi pain.

**Conclusions:**

The mid‐term clinical and radiographic results were satisfactory in patients who underwent the subtalar arthroereisis procedure using Talar‐Fit implant, alone or in combination with other adjuncts, for the treatment of flexible flatfoot.

## Graphical abstract

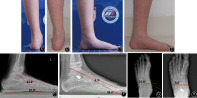

The mid‐term clinical and radiographic results were satisfactory in flexible flatfoot children and adolescents who underwent subtalar arthroereisis, alone or in combination with other adjunctive procedures. Implant position was associated with postoperative sinus tarsi pain.

## Introduction

Flexible flatfoot (FFF) is a common disease with a reported incidence of 5% in children and adults. The main characteristics of FFF are collapse of the medial longitudinal arch, hindfoot valgus, and forefoot abduction caused by excessive eversion of the subtalar joint, and most patients are asymptomatic^1^. Other characteristics are usually observed, such as contracture of the Achilles tendon or the gastrocnemius aponeurosis, spasm of the peroneus, and medial column instability. Although the treatment of FFF is still controversial^2^, surgery is appropriate for patients with significant pain along the medial side of the foot, easy fatigue, gait changes, and compromised ankle dorsiflexion^3^.

The surgical procedures can be categorized as tendon lengthening and transfers, osteotomies, subtalar arthroereisis (STA), and arthrodesis of one or more joints. Isolated soft tissue procedures routinely lead to unsatisfactory outcomes and are combined with other procedures in most cases^4^. Osteotomies include medial displacement calcaneal osteotomy (MDCO) and lateral column lengthening (LCL), and both types are capable of correcting valgus deformity of the hindfoot. However, they do not actually address the deformity of the subtalar joint complex, and patients are faced with the risk of nonunion or malunion and a longer recovery time. Arthrodesis should be avoided if at all possible because of the loss of the shock‐absorbing function of the subtalar joint complex and the tendency to develop early degenerative arthritis in adjacent joints^4^, ^5^, ^6^. STA refers to the implantation of a device in the sinus tarsi to restrict excessive eversion of the subtalar joint and consequently correct the flatfoot deformity. At present, it has been widely used clinically, especially in children and adolescents with FFF. Its advantages include being minimally invasive, providing a three‐dimensional correction, and its rapid recovery^7^. However, STA still remains a controversial procedure as the evidence is poor and based mainly on cohort studies with a small sample and a short‐term follow‐up^8^, ^9^.

In clinical practice, STA is often accompanied by other procedures to achieve complete correction of the deformity, such as gastrocnemius recession or Achilles tendon lengthening, resection of the accessory scaphoid, reconstruction of the posterior tibialis tendon, and even medial column arthrodesis^10^. The effects of adjunctive procedures on the functional and radiographic outcomes have rarely been studied in the literature. Cicchinelli *et al*. conducted a retrospective analysis of pediatric FFF patients to identify the role of arthroereisis, alone or in combination with other adjunctive procedures, for radiographic outcomes. The authors found that gastrocnemius recession had a positive effect on the correction of transverse plane deformity when used as an adjunct to STA and medial column arthrodesis had a negative impact on the degree of correction in the transverse plane as an adjunct to STA and gastrocnemius recession^11^. However, their sample size was small and they did not measure foot‐related quality of life, pain, or other important clinical outcomes. Thus, evidence is still lacking with regard to the role of arthroereisis alone or in association with other adjunctive procedures for correction of the flatfoot deformity.

Sinus tarsi pain is the most common postoperative complication of STA with a reported incidence of approximately 10% to 40%^7^, and many patients require implant removal or replacement to address this issue. The etiology of postoperative sinus tarsi pain is multifactorial and not fully known. Cook *et al*. performed a propensity‐matched case–control study to identify risk factors in subtalar arthroereisis explantation and found that patients who required implant removal had greater odds of radiographic undercorrection^12^. Their results suggested that higher postoperative anteroposterior (AP) talar‐first metatarsal angles and calcaneocuboid abduction angles were associated with greater odds of undergoing implant removal, and that smaller postoperative AP Kite angles were associated with a 16.7% reduction in odds for removal. Patient age, gender, implant size, shape, position, and adjunctive procedures were found to be insignificant factors. Saxena *et al*. performed a prospective study to determine the risk factors for removal of the implant caused by sinus tarsi pain in adults treated for adult acquired flatfoot deformity or posterior tibial tendon dysfunction^13^. Contrary to the results of Cook *et al*., they found that implant size was a risk factor for explanation, with 11‐mm implants removed most frequently, while gastrocnemius recession and patient age were not. Although other variables such as overcorrection, soft tissue irritation, and impingement between the screw and the posterior subtalar articular surface have also been suspected as possible sources of pain^12^, ^14^, ^15^, the actual risk factors are not yet clear.

Therefore, the purposes of this study are to: (i) report the mid‐term outcomes of STA using Talar‐Fit implant for the treatment of pediatric FFF patients; (ii) compare clinical and radiographic outcomes between arthroereisis with and without adjunctive operative procedures to investigate the effects of adjuncts on the outcomes; and (iii) analyze the risk factors associated with sinus tarsi pain, which is the most common postoperative complication of arthroereisis.

## Patients and Methods

### *Inclusion and Exclusion Criteria*

Inclusion criteria included: (i) patients diagnosed as FFF and aged between 9 and 20 years at the time of operation; (ii) underwent STA in our hospital between June 2014 to May 2019; (iii) a successful follow‐up to enable comparison between preoperative and postoperative measurements; (iv) clinical and radiographic outcomes were accessible; and (v) a retrospective study. Exclusion criteria were: (i) patients lost to follow‐up; (ii) rigid flatfoot; (iii) neurological flatfoot; and (iv) patients with previous surgery for the treatment of FFF.

### *Prosthesis*

**Fig 1:**
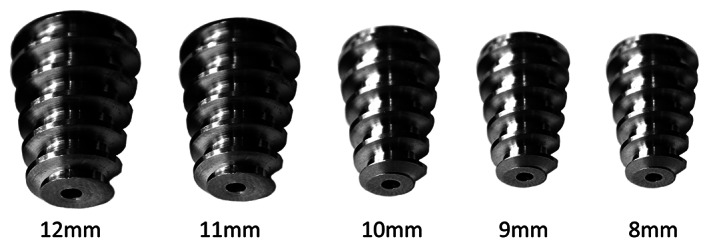
The Talar‐Fit implants (size 12, 11, 10, 9, and 8 mm).

The titanium alloy Talar‐Fit (Osteomed, Addison, TX, USA) implant was employed in all patients (Fig. 1). It adopts a conical shape to adapt to the anatomical features of the sinus tarsi. Deep and blunt threads are claimed to promote soft tissue ingrowth and reduce irritation. It is categorized as type IB self‐locking wedge device according to Graham's classification^16^.

### *Surgical Techniques*

#### *Adjunctive Procedures*

**Fig 2:**
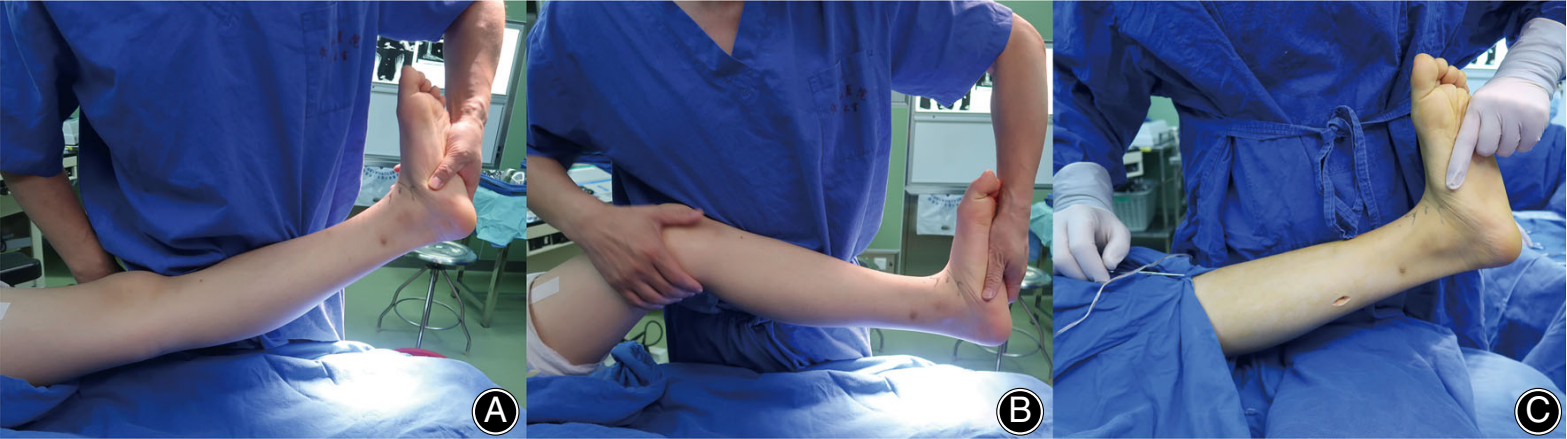
An illustration of pre‐ and postoperative Silfverskiold tests. (A) Preoperative test showed that the amount of ankle dorsiflexion was less than 10° with the subtalar joint locked in inverted position and the knee extended. (B) The ankle joint could be dorsiflexed more than 10° with the subtalar joint locked in inverted position and the knee flexed, indicating gastrocnemius contracture. (C) After gastrocnemius recession, Silfverskiold test was rechecked and the amount of ankle dorsiflexion was more than 10° with the subtalar joint inverted and the knee extended.

The patients were placed in a supine position and general anesthesia or continual epidural anesthesia was used. First, we assessed whether the patients were accompanied by Achilles tendon contracture or gastrocnemius contracture before surgery through the Silfverskiold test^17^. If the ankle joint could be dorsiflexed more than 10° with the subtalar joint locked in inverted position and the knee flexed, but less than 10° with the knee extended, then gastrocnemius contracture was indicated and a gastrocnemius recession was performed^3^, ^17^. A 4‐ to 5‐cm longitudinal posteromedial incision was made approximately midway between the knee and the ankle, the sural nerve and the long saphenous vein were protected, and the musculotendinous junction of the gastrocnemius was identified. After careful dissection, the gastrocnemius aponeurosis was cut as far distally as possible and the plantaris tendon was divided. Finally, we rechecked the Silfverskiold test to ensure the amount of ankle dorsiflexion was more than 10° with the subtalar joint inverted and the knee extended (Fig. 2). If the ankle joint was dorsiflexed less than 10° with the subtalar joint locked in inverted position and the knee both extended and flexed preoperatively, it indicated Achilles tendon contracture; then, a triple‐hemisection percutaneous Achilles tendon lengthening would be performed (Hoke technique)^18^.

If the accessory scaphoid was present in preoperative X‐rays and there was a significant tenderness at the location of the accessory scaphoid during physical examination, we would perform dissection of the accessory scaphoid and reconstruction of the end point of the posterior tibialis tendon (Kidner procedure)^19^. If the tenderness was negligible, the accessory scaphoid would not be removed and the Kidner procedure would be omitted. In addition, Cotton osteotomy was performed in cases of significant medial column instability^20^.

#### *Subtalar Arthroereisis*

**Fig 3:**
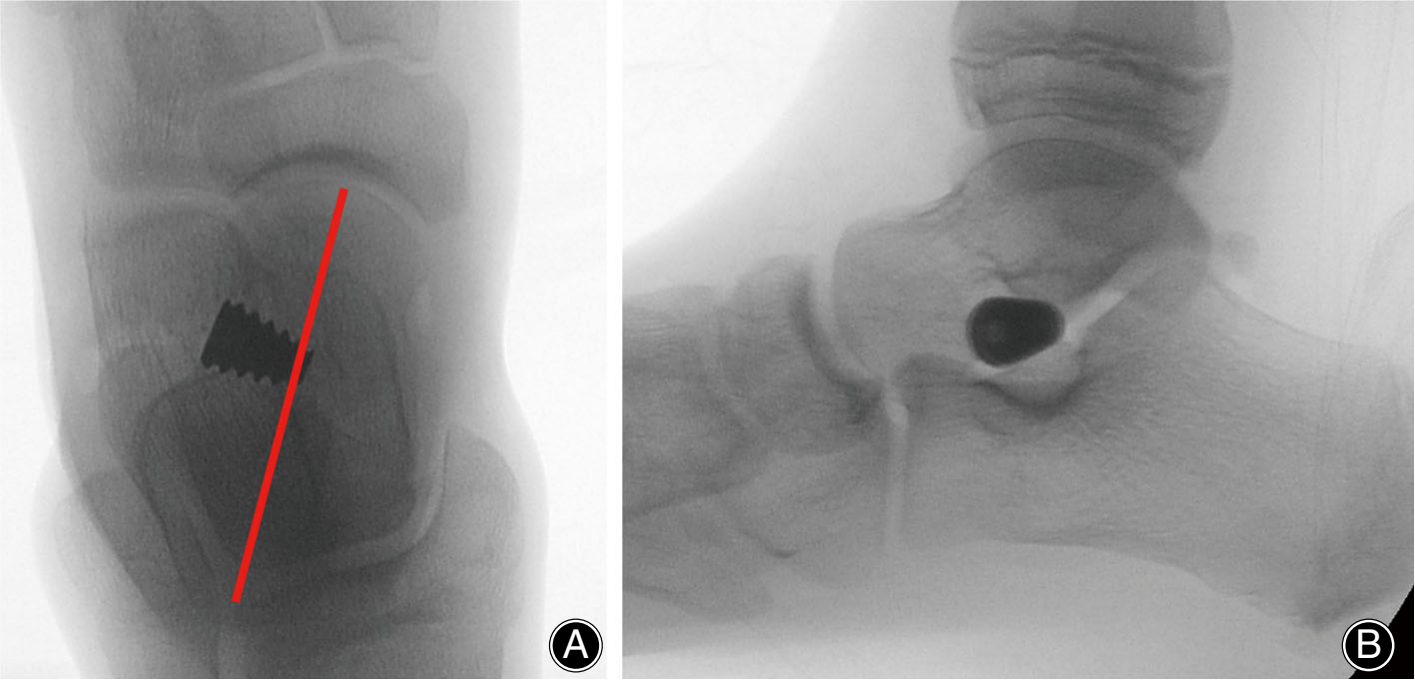
Typical intraoperative X‐ray films after the permanent implant was inserted, showing that this device was inserted in an anterior‐lateral to posterior‐medial orientation. (A) Anteroposterior view showing that the leading edge slightly exceeded the talar bisection line; and (B) lateral view.

The tarsal sinus was approached through a 1‐ to 2‐cm slightly curved incision. The subcutaneous tissue and the deep fascia were bluntly dissected to expose the tarsal sinus. Then the Talar‐Fit guide pin was inserted in an anterolateral‐distal to posteromedial‐proximal orientation, passing through the tarsal canal. A small incision was made to allow passage of the guide pin through the medial aspect of the foot. The trial implants were inserted with the subtalar joint inverted. The appropriate size would allow a good screw purchase and the calcaneal subtalar joint complex to evert to approximately 2° to 4°, with the tail end of the implant 1 to 1.5 cm beyond the lateral calcaneal wall. It should be noted that although the Talar‐Fit instructions recommended the location of the implant within the sinus portion of the tarsal sinus and the leading edge not exceeding the talar bisection line, we chose the location within the canalis portion to increase stability (Fig. 3). Finally, the appropriate permanent implant was inserted. The implant position was checked again with fluoroscopy, and ROM of the subtalar joint was examined to be physically normal. Incision was closed in layers, and a compression dressing was applied.

#### *Postoperative Management*

All patients received short leg cast immobilization for 6 weeks after operation to maintain the neutral position of the ankle joint and avoid weight‐bearing. The cast was removed after 6 weeks and weight‐bearing was allowed gradually after its removal.

### *Clinical Evaluation*

#### *American Orthopaedic Foot and Ankle Society (AOFAS) Ankle and Hindfoot Score*

The AOFAS ankle and hindfoot score was used to evaluate postoperative recovery of ankle and hindfoot function for FFF patients. It mainly includes three aspects: pain, function, and alignment. The score standard had a maximum of 100 points. A mark of 90–100 was considered as excellent, 75–89 as good, 50–74 as fair, and <50 as poor^21^. Postoperative complications were recorded including pain, dislocation^22^, and revision.

### *Radiographic Measurement*

For radiographic evaluation, the following angles were measured on both pre‐ and postoperative weight‐bearing radiographs to perform quantitative comparisons^23^, ^24^.

#### *The Lateral Talar‐First Metatarsal (Meary's) Angle*

**Fig 4:**
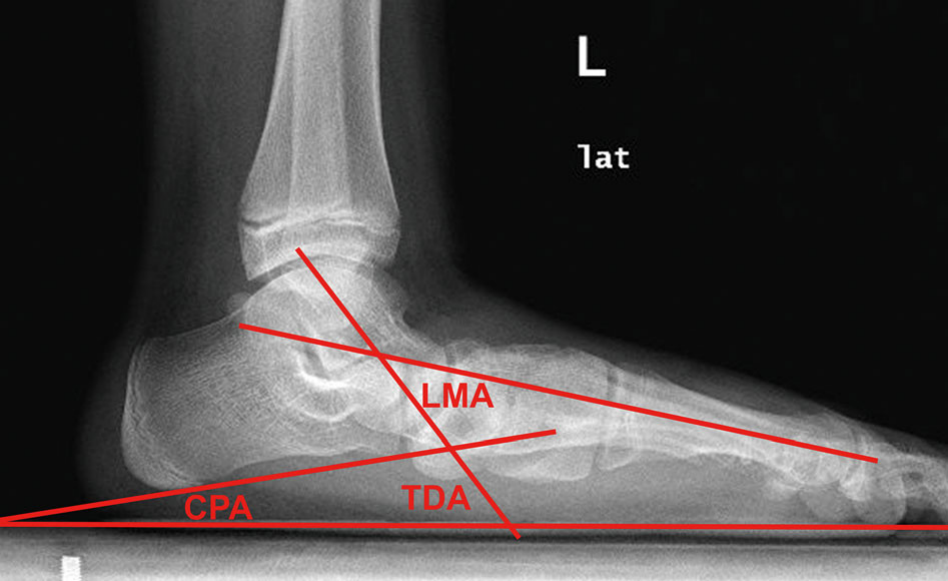
Lateral view of preoperative radiograph, illustrating measured angles. CPA, calcaneal pitch angle; LMA, lateral Meary's angle; TDA, talar declination angle.

The lateral Meary's angle was measured as the angle formed between the longitudinal axis of the talus and the axis of the first metatarsal (negative values were noted if the axis of the first metatarsal was oriented in dorsiflexion) (Fig. 4).

#### *The Calcaneal Pitch Angle*

The calcaneal pitch angle was defined as the angle formed by a line parallel to the ground and a line connecting the two most inferior points on the calcaneus on the lateral view^25^ (Fig. 4).

#### *The Talar Declination Angle*

The angle formed between the longitudinal axis of the talus and a line parallel to the ground on the lateral view (Fig. 4).

#### *The AP Talar‐First Metatarsal (Meary's) Angle*

**Fig 5:**
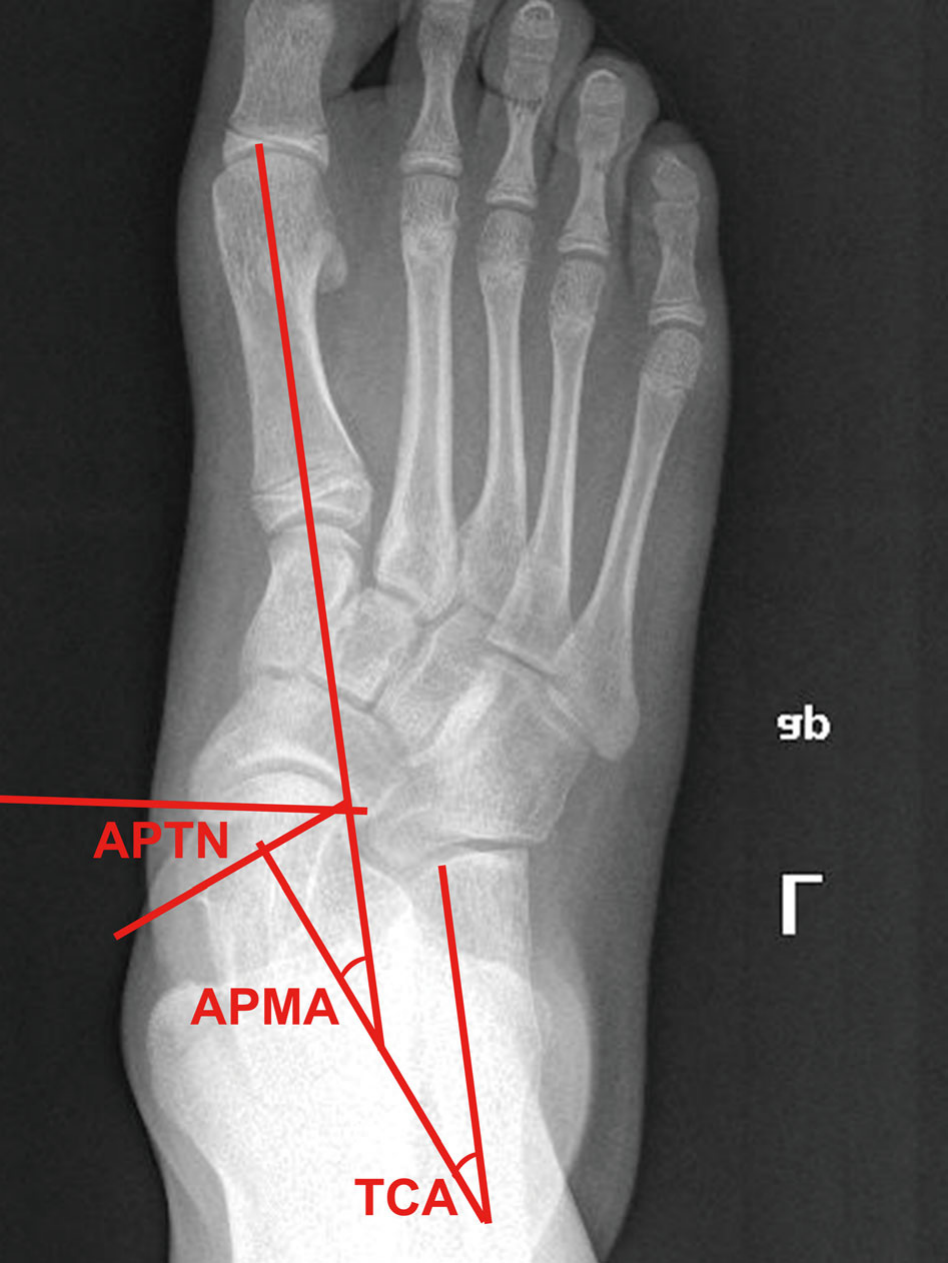
Anteroposterior view of preoperative X‐ray, illustrating measured angles. APMA, anteroposterior Meary's angle; APTN, anteroposterior talonavicular coverage angle; TCA, talocalcaneal angle.

Similar to the lateral Meary's angle, the AP Meary's angle was traced between the long axis of the talus and the axis of the first metatarsal on the AP view (Fig. 5).

#### *The AP Talocalcaneal Angle*

The AP talocalcaneal angle was measured as the angle between the axis of the talus and the axis of the calcaneus on the AP view (Fig. 5).

#### *The AP Talonavicular Coverage Angle*

The angle formed between the lines connecting the endpoints of the talar and navicular articular surfaces (Fig. 5).

**Fig 6:**
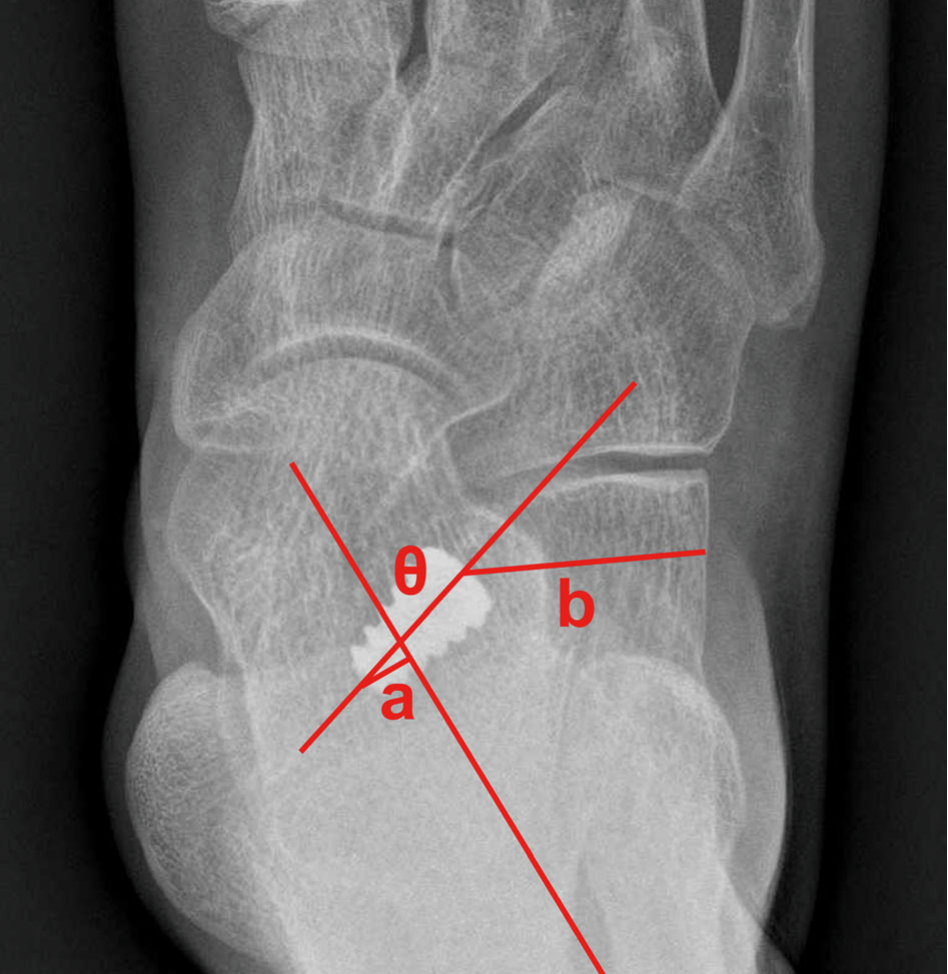
Anteroposterior view of postoperative X‐ray, illustrating the definitions of implant depth (line segment a), position (line segment b), and orientation (angle θ).

In addition, the implant depth, position, and orientation were measured on the AP X‐rays. The implant depth was defined as the perpendicular distance from the leading edge of the implant to the longitudinal talar bisection line (negative values were noted if the leading edge did not exceed the talar bisection), the implant position as the perpendicular distance from the tail end of the device to the lateral calcaneal wall, and the implant orientation as the angle formed between the longitudinal axis of the implant and the talar bisection^12^ (Fig. 6).

### *Statistical Analysis*

The results of AOFAS score and radiographic angles were presented as mean and standard deviation (SD). SPSS 23.0. (IBM, Armonk, NY, USA) was used for data analysis. The paired Student's *t*‐test was used to compare the pre‐ and postoperative angular measurements and AOFAS scores. The Wilcoxon rank‐sum test was undertaken to determine the outcome differences among four treatment groups: STA alone, STA with gastrocnemius recession, STA with Kidner procedure, and STA with gastrocnemius recession and Kidner procedure. Multivariate logistic regression analysis was used to analyze risk factors for sinus tarsi pain. Regression candidates included age at the time of initial surgery, gender, implant size, follow‐up duration, pre‐ and postoperative angular measurements, and implant depth, position, and orientation. *P* value <0.05 is considered statistically significant.

## Results

### *Follow‐up and General Results*

A total of 31 patients (46 feet) were included in this study. Of the 31 patients, 26 (83.9%) were male and five (16.1%) were female, with a mean age at the time of surgery of 12.8 years (range, 11–20 years). The right foot was involved in 22 (47.8%) of the feet and the left foot was involved in 24 (52.2%). The mean follow‐up of the feet was 32.8 months (range, 10–71 months).

**Fig 7:**
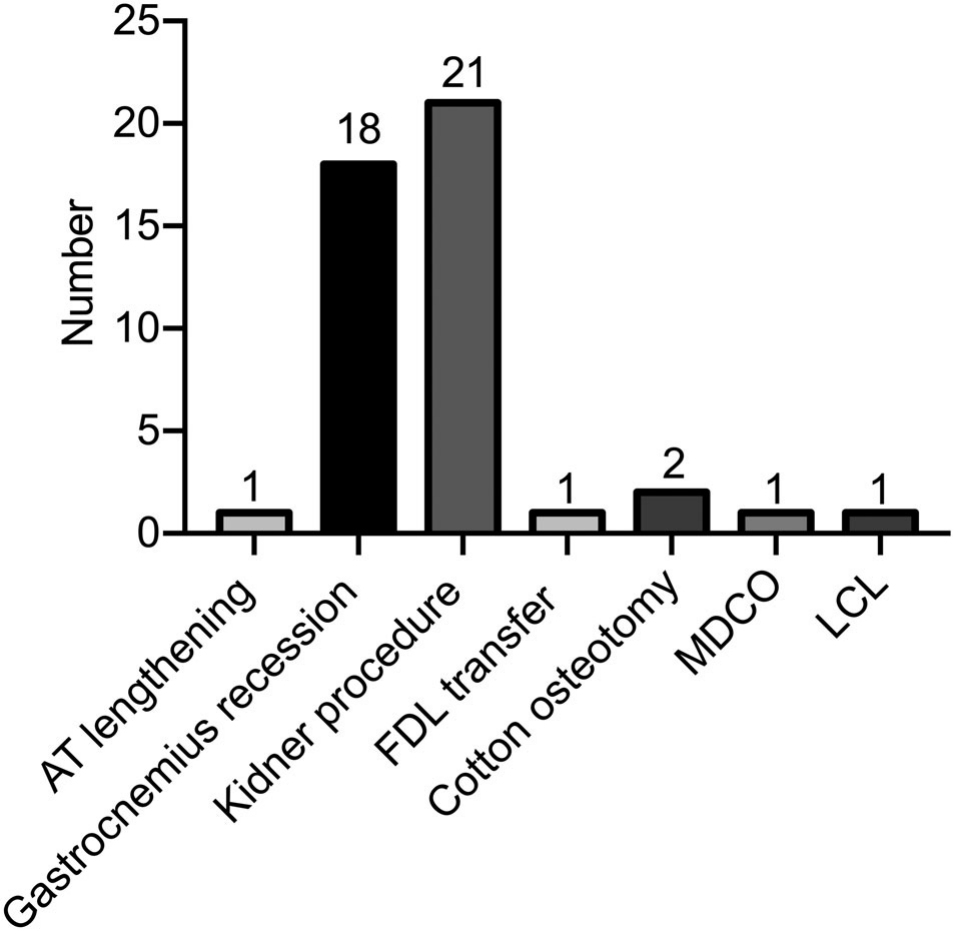
Number of adjunctive procedures performed. AT, Achilles tendon; FDL, flexor digitorum longus; LCL, lateral column lengthening; MDCO, medial displacement calcaneal osteotomy.

The mean operation time was 46.1 min (range, 30–72 min), and mean blood loss was 17.0 mL (range, 5–30 mL). Intraoperatively, increased ROM of the subtalar joint was confirmed in all feet. STA was performed alone in 10 feet (21.7%). The surgical techniques most often associated with arthroereisis were gastrocnemius recession (18 feet, 39.1%) and Kidner procedure (21 feet, 45.7%) (Fig. 7).

To determine the effects of adjunctive procedures on the outcomes, the feet were divided into four groups: STA alone (*n* = 10), STA with gastrocnemius recession (*n* = 12), STA with Kidner procedure (*n* = 13), and STA with gastrocnemius recession and Kidner procedure (*n* = 6). There were no significant differences among the four groups with regard to the baseline characteristics except for follow‐up period (*P* = 0.046) (Table 1).

**TABLE 1:** Comparison of baseline characteristics among four treatment subgroups (mean ± SD)

Characteristics	†Group 1	‡Group 2	§Group 3	¶Group 4	*P*‐value
Age at the time of surgery (years)	12.1 ± 0.6	13.8 ± 3.2	12.3 ± 1.0	12.9 ± 0.7	0.240
Follow‐up period (months)	23.7 ± 12.0	27.4 ± 15.6	43.8 ± 23.0	19.9 ± 20.7	0.046*
Height (cm)	168.4 ± 9.1	168.6 ± 7.1	166.0 ± 12.1	166.0 ± 9.7	0.853
Weight (kg)	59.3 ± 13.3	53.6 ± 11.2	55.0 ± 8.7	53.5 ± 6.8	0.975
AOFAS score	53.6 ± 19.2	59.8 ± 8.7	53.4 ± 14.8	61.5 ± 8.5	0.863
Preoperative lateral view (°)					
Meary's	−17.5 ± 11.8	−24.3 ± 11.0	−13.0 ± 11.3	−21.1 ± 9.1	0.171
Calcaneal pitch	15.8 ± 3.5	17.4 ± 4.1	15.8 ± 6.5	12.7 ± 5.1	0.353
Talar declination	36.9 ± 9.3	40.5 ± 8.5	31.1 ± 9.5	36.4 ± 8.2	0.252
Preoperative AP view (°)					
Meary's	15.1 ± 8.1	21.4 ± 7.4	18.4 ± 9.6	27.1 ± 9.9	0.114
Talocalcaneal	24.5 ± 5.4	28.0 ± 7.6	21.8 ± 4.6	25.7 ± 7.7	0.240
Talonavicular coverage	15.1 ± 6.5	21.5 ± 5.1	17.4 ± 9.3	24.6 ± 10.2	0.074

AOFAS, American Orthopaedic Foot and Ankle Society; AP, anteroposterior.

* Significant.

^†^ Group 1, STA alone;

^‡^ Group 2, STA with gastrocnemius recession,

^§^ Group 3, STA with Kidner procedure;

^¶^ Group 4, STA with gastrocnemius recession and Kidner procedure.

### *Radiographic and Clinical Outcomes*

**Fig 8:**
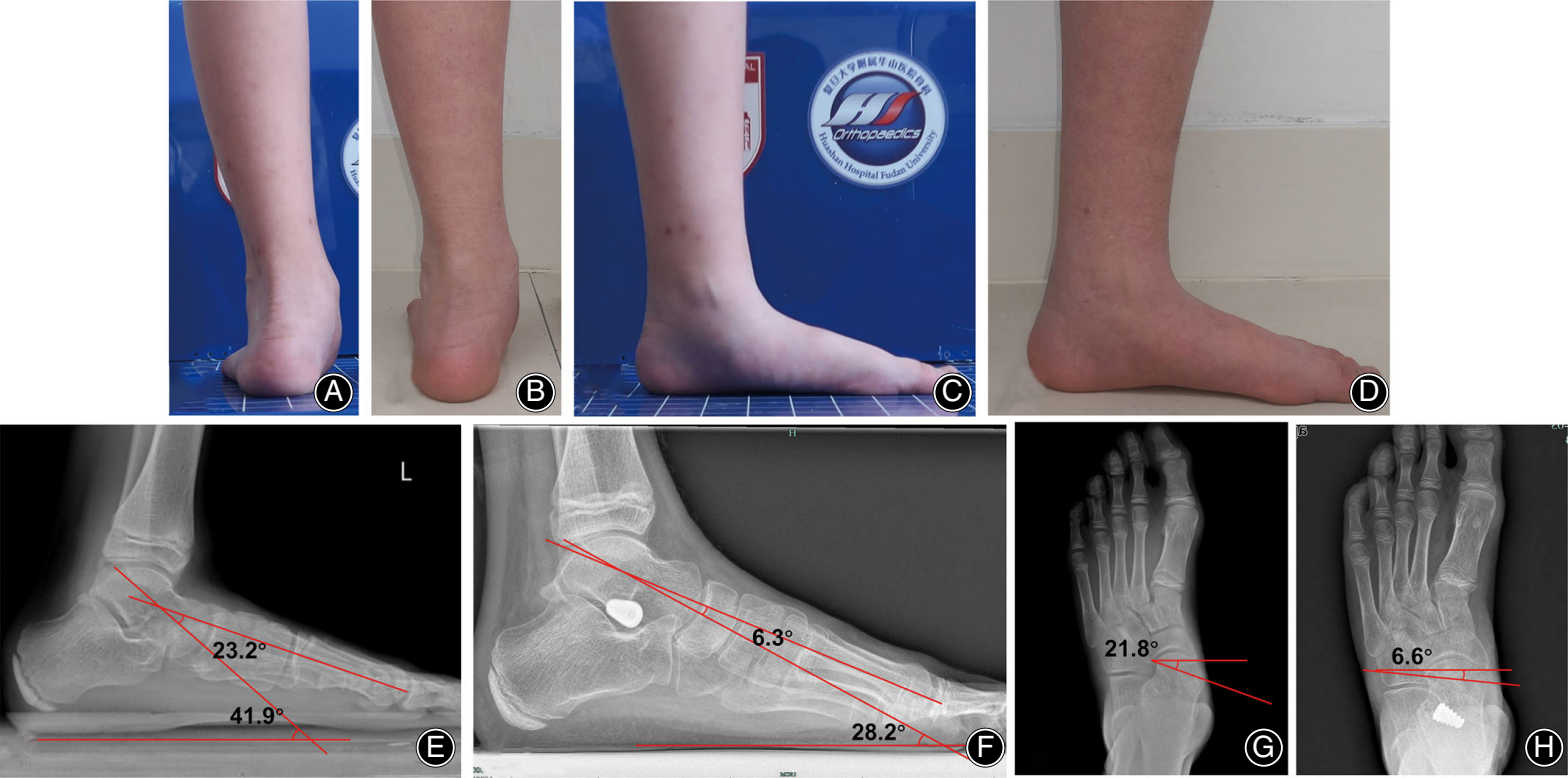
Gross photos and X‐ray films of the left foot before and 1 year after surgery in a typical patient (male, 12 years old at the time of surgery). (A, B) Comparison of gross photos of hindfoot before and 1 year after surgery showing that the hindfoot valgus deformity was corrected; (C, D) comparison of gross photos of the medial aspect of the foot before and 1 year after surgery showing appearance of the medial longitudinal arch; (E) lateral weight‐bearing X‐ray before surgery showing lateral Meary's angle was −23.2° and talar declination angle was 41.9°; (F) lateral weight‐bearing X‐ray 1 year after surgery showing lateral Meary's angle decreased to −6.3° and talar declination angle decreased to 28.2°; (G) AP X‐ray before surgery showing AP talonavicular coverage angle was 21.8°; and (H) AP X‐ray 1 year after surgery showing AP talonavicular coverage angle decreased to 6.6°. AP, anteroposterior.

Comparison of radiographic outcomes showed that the lateral talar‐first metatarsal (Meary's) angle decreased by a mean of 19.1° ± 11.9° (*P* < 0.001), the calcaneal pitch angle increased by a mean of 5.4° ± 3.4° (*P* < 0.001), the talar declination angle decreased by a mean of 14.8° ± 9.9° (*P* < 0.001), the AP talar‐first metatarsal (Meary's) angle decreased by a mean of 15.6° ± 10.3° (*P* < 0.001), the talocalcaneal angle decreased by a mean of 7.2° ± 8.3° (*P* = 0.001), and the talonavicular coverage angle decreased by a mean of 20.4° ± 9.0° (*P* < 0.001) (Table 2, Fig. 8). The mean AOFAS score significantly improved from 55.5 ± 14.5 (range, 24–74) preoperatively to 86.3 ± 9.9 (range, 60–97) at the final follow‐up (*P* < 0.001).

**TABLE 2:** Radiographic comparison between preoperative and last follow‐up values in feet treated with subtalar arthroereisis (mean ± SD)

Angles	Preoperative	Last follow‐up	*P*‐value	Change
Lateral view (°)				
Talar‐first metatarsal (Meary's)	−24.4 ± 10.4	−5.3 ± 5.5	0.000	19.1 ± 11.9
Calcaneal pitch	13.5 ± 5.4	18.9 ± 5.3	0.000	5.4 ± 3.4
Talar declination	40.2 ± 8.5	25.4 ± 4.4	0.000	14.8 ± 9.9
Anteroposterior view (°)				
Talar‐first metatarsal (Meary's)	22.1 ± 9.1	6.6 ± 7.4	0.000	15.6 ± 10.3
Talocalcaneal	25.9 ± 9.2	18.2 ± 6.8	0.001	7.2 ± 8.3
Talonavicular coverage	23.3 ± 7.9	3.3 ± 7.3	0.000	20.4 ± 9.0

### *Subgroup Analysis*

Table 3 showed the clinical and radiographic correction obtained in the four treatment groups, namely, STA alone (group 1), STA with gastrocnemius recession (group 2), STA with Kidner procedure (group 3), and STA with gastrocnemius recession and Kidner procedure (group 4), for the AOFAS score and different angles measured. There were no statistically significant differences with regard to AOFAS score, lateral Meary's angle, calcaneal pitch angle, talar declination angle, AP talocalcaneal and AP talonavicular coverage among the four groups (*P* > 0.05). Although the overall difference for AP Meary's angle was significant (*P* = 0.046), all adjusted *P*‐values were greater than 0.05 after pairwise comparisons across groups (Table 3).

**TABLE 3:** Comparison of clinical and radiographic outcomes at last follow‐up among four treatment subgroups (mean ± SD)

Outcomes	†Group 1	‡Group 2	§Group 3	¶Group 4	*P*‐value
AOFAS score	87.4 ± 9.6	90.8 ± 4.6	81.0 ± 15.9	88 ± 4.1	0.772
Lateral view angles (°)					
Meary's	−5.2 ± 4.8	−1.9 ± 4.4	−6.2 ± 6.3	−1.7 ± 2.4	0.585
Calcaneal pitch	17.6 ± 2.8	20.8 ± 1.8	18.2 ± 8.7	20.3 ± 4.4	0.640
Talar declination	25.4 ± 4.7	23.1 ± 3.9	27.0 ± 4.0	20.3 ± 2.2	0.252
AP view angles (°)					
Meary's	−2.7 ± 1.7	11.3 ± 4.8	7.3 ± 6.6	−2.0 ± 9.1	0.046*
Talocalcaneal	10.1 ± 5.9	22.6 ± 6.3	16.3 ± 4.3	9.3 ± 5.4	0.054
Talonavicular coverage	−6.3 ± 2.2	8.4 ± 7.2	2.6 ± 5.1	−1.2 ± 4.9	0.071

AOFAS, American Orthopaedic Foot and Ankle Society; AP, anteroposterior.

* Although the overall difference was significant, all adjusted *P‐*values were greater than 0.05 after pairwise comparisons across groups.

^†^ Group 1, STA alone;

^‡^ Group 2, STA with gastrocnemius recession,

^§^ Group 3, STA with Kidner procedure;

^¶^ Group 4, STA with gastrocnemius recession and Kidner procedure.

### *Complications*

**Fig 9:**
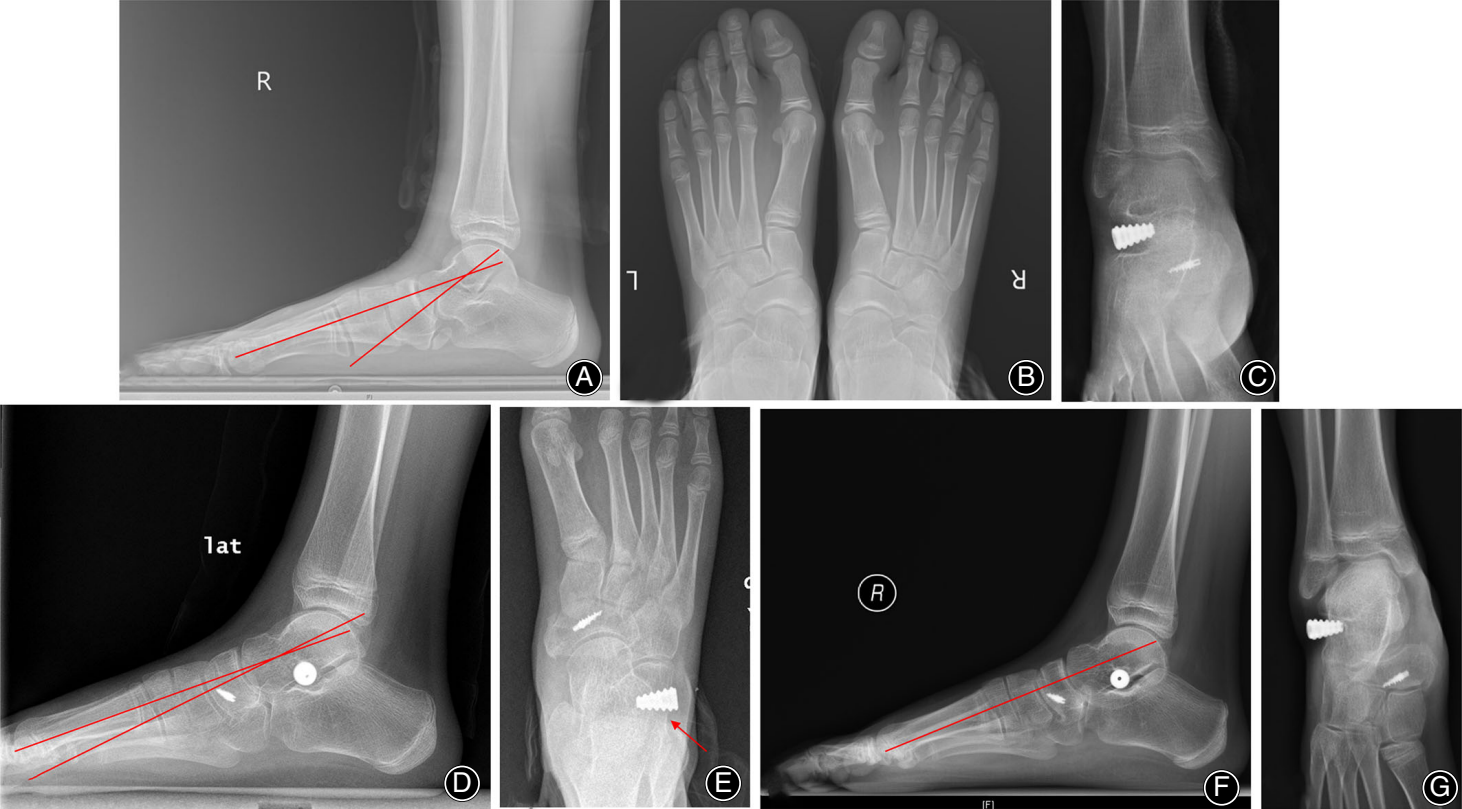
X‐ray films of the dislocation case (female, 11 years old at the time of surgery, right foot). (A, B) Preoperative lateral and anteroposterior weight‐bearing X‐rays showed the flatfoot deformity; (C) Dislocation was caused by a fall 6 weeks after surgery and confirmed by X‐ray; (D, E) Nonoperative treatment was administrated; lateral and anteroposterior weight‐bearing X‐rays 6 months after surgery showed that the flatfoot deformity was corrected although the implant was in dislocated position (the tail end of the implant lateral to the lateral calcaneal wall (Arrow)); (F) Eighteen months after surgery, lateral weight‐bearing X‐ray showed that the correction was maintained; (G) Anteroposterior X‐ray 18 months after surgery showed that the device was dislocated more laterally; the implant could be palpable in the sinus tarsi area.

Six feet (13%) complained of the presence of pain in the sinus tarsi, requiring implant removal in one foot. There was one dislocation case, which was caused by a fall six weeks after surgery and then confirmed by X‐ray. She underwent nonoperative treatment and took two more weeks to start weight‐bearing. At the final follow‐up, she had no symptoms and had resumed all daily activities although the implant could be palpable in the sinus tarsi area (Fig. 9).

### *Multivariate Logistic Regression*

Age, implant size, follow‐up duration, angular measurements, and implant depth, position, and orientation were analyzed as continuous covariates. Logistic regression analysis showed that implant position was the only risk factor associated with sinus tarsi pain with an odds ratio of 1.388 (*P* = 0.025, 95% *CI*: 1.042–1.849). In other words, patients with a longer distance from the tail end of the implant to the lateral calcaneal wall had 38.8% greater odds of developing sinus tarsi pain.

## Discussion

The treatment of FFF with STA is still controversial. In this study, we reported the mid‐term results of STA using Talar‐Fit implant for the treatment of FFF, investigated the effects of adjunctive procedures on the outcomes, and analyzed the risk factors associated with postoperative pain in the sinus tarsi area.

### *Role of STA in the Treatment of FFF and Comparison Between STA and Other Procedures*

Compared with traditional osteotomies such as MDCO and LCL, STA has many advantages: the procedure is easy and less invasive; and there is no problem of nonunion or malunion since no osteotomy is performed, so it requires less time to recover. In addition, unlike MDCO which can only correct the flatfoot deformity on the coronal plane (hindfoot valgus) and LCL on the transverse plane (forefoot abduction), STA provides a three‐dimensional correction by preventing the talus from slipping forward, inward, and downward during pronation^7^. Finally, it does not affect the bone development and does not interfere with potential osteotomies that may be needed in the future.

Fernández *et al*. argued that STA was an alternative to MDCO for the correction of valgus hindfoot in FFF patients but was not suitable for forefoot abduction deformity^7^. There are few controlled studies comparing these procedures and the level of evidence is low. Chong *et al*. performed a prospective study comparing STA with LCL for the treatment of pediatric FFF^26^. At 1‐year follow‐up, they did not find statistically significant differences between the two groups with regard to clinical, radiographic, and kinematic outcomes. However, the groups were not randomized and the LCL group had a greater preoperative radiographic deformity. In summary, randomized controlled trials (RCTs) need to be done in many specific areas to determine the role of STA in the treatment of FFF and which procedure is superior.

Graham *et al*. reported the 5‐year functional outcomes of 83 adult FFF patients treated with the self‐locking wedge implant and found that the mean postoperative Maryland Foot Score was 88 out of 100 and 80% of the patients were satisfied with the appearance of their feet^27^. The mean talar second metatarsal angle decreased by 19°, the mean talar declination angle decreased by 5.7°, and the mean calcaneal pitch angle increased by 0.8°^28^. De Pellegrin *et al*. conducted a retrospective study of 485 FFF children treated with the calcaneo‐stop implant. The average follow‐up was 4.5 years and 93.7% of cases reported satisfactory clinical and radiographic outcomes without complications. The mean talar declination angle decreased by 18° and the mean calcaneal pitch angle increased by 3°^29^. These results were comparable to those of our study.

### *Effects of Adjunctive Procedures on the Outcomes*

STA is often performed in combination with other procedures in order to achieve full correction. Research regarding the effects of adjunctive procedures is lacking. Cicchinelli *et al*. undertook a retrospective evaluation of pediatric pes valgus patients who had undergone STA as a sole intervention, or in combination with other adjuncts, and suggested that gastrocnemius recession displayed a positive effect on the correction of transverse plane deformity when used as an adjunct to STA, and medial column arthrodesis had a negative impact as an adjunct to STA and gastrocnemius recession^11^. However, our findings did not find the effects of gastrocnemius recession on both clinical and radiographic outcomes and medial column arthrodesis was not taken into account because it was performed on only two feet (4.3%). Further studies are needed due to the small sample size of both studies.

### *Risk Factors Associated with Sinus Tarsi Pain*

Sinus tarsi pain is the most common complication with a considerably high rate (10%–40%)^7^. The pathogenesis of sinus tarsi pain is not completely understood. Overcorrection, undercorrection, impingement between the screw and the posterior subtalar articular surface, and soft tissue irritation have been considered as possible causes^9^, ^10^, ^11^. Saxena *et al*. performed a prospective study to determine the risk factors for removal of the implant caused by sinus tarsi pain in adults treated for adult acquired flatfoot deformity or posterior tibial tendon dysfunction and found that implant size was a factor for removal, with 11‐mm implants explanted most frequently, while gastrocnemius recession and patient age were not^13^. Our finding suggested that implant position was also a risk factor and patients with a longer distance from the tail end of the implant to the lateral calcaneal wall had 38.8% greater odds of developing sinus tarsi pain. Therefore, we recommend that when both a smaller size with a longer distance from the tail end of the implant to the lateral calcaneal wall and a bigger size with a shorter distance could achieve the satisfactory correction, choosing the bigger one could reduce the incidence of postoperative sinus tarsi pain.

### *Limitations of the Study*

We acknowledge that this study has limitations, and its results should be understood with these limitations in mind. First, it was retrospective and may be biased, such as selection bias and information bias. Second, the evaluation of STA could have been compared with patients treated with nonoperative methods and our study lacked a control group. In addition, although the overall sample size was not small, we only had a small number of feet in each treatment group, which may affect the reliability of our results. Future prospective, randomized controlled studies are needed to confirm our results. Finally, aside from the factors in our logistic regression model, there may be other significative factors such as the shape and material type of the implant; thus, our results may have changed if these factors were considered.

### *Conclusion*

In conclusion, the present study indicated that the mid‐term clinical and radiographic results were satisfactory in patients who underwent the subtalar arthroereisis procedure using Talar‐Fit implant, alone or in combination with other adjuncts, for the treatment of flexible flatfoot. Implant position was associated with postoperative sinus tarsi pain. Further research is needed to provide the long‐term outcomes, and RCTs need to be done in many specific areas around flexible flatfoot.
